# Modern Sociology

**Published:** 1900-08-11

**Authors:** 


### Modern Sociology.

### THE CHILD-WORKER.

The committee which investigated the circumstances of wage-earning children of school-age have published a summary of their opinions on the point in a pamphlet which may be obtained from the hon. secretary, Mrs. F. G. Hogg, at a price of 1d. each, or 5s. a 100. The pamphlet does not give all the cases in which the committee found out that children who were at school during one part of the day were, for great part of the remainder, employed in work so laborious that it rendered them unfit to profit by the educational opportunities within their reach. Those who read the pamphlet have had, it is presumed, an opportunity of acquainting themselves with the facts in full, as discovered by the committee; but enough cases are given to justify the truth of Sir John Gorst's opinion that the whole report is "a sickening document." Take a few samples: A boy of 6 delivers milk, probably before school, for 28 hours, and a girl of 5 for 35 hours a week; a boy of 10 works on a farm for 72 hours, another of 12 in a chemist's shop for 78 hours a week; a newspaper boy sells papers for 100 hours a week; another is out every morning between three and four o'clock, to call workmen, after which he delivers papers from half-past five to nine o'clock a.m., when his school work is supposed to begin; another works four whole nights a week in a dust-yard, from nine p.m. to six a.m.

Cases like these it is difficult for the law to touch. The law can enforce school attendance, but how can it prevent work outside of school hours, even though it is plain, as it is in the cases quoted, that the work is so prolonged and of so exhausting a nature (relative to the age and strength of the worker, and the demands made on his energies by the requirements of education) that his schooling will profit him nothing? The Education Act of 1876 indeed set out with the intention of protecting children of school age from labour. Section 5 states that "a person shall not take into his employment any child," and a "child" is further defined as "a child between the ages of 5 and 14 years." Anyone taking a child into his employment in contravention of this Act is liable to a penalty of 40 shillings. Moreover, section 47 enacts that "a parent of a child who employs such child in any labour exercised by way of trade, or for the purposes of gain, shall be deemed to take such child into his employment."

This enactment seems to be as complete and satisfactory as it could be made. But, unfortunately, after laying down this excellent principle, the Act proceeded to name a number of cases in which exemption from its operations would be granted. Some of these have since been overridden by the Act of 1880, but the remainder still exist to hamper the work of education, and encourage selfish parents, and magistrates and school attendance committees, who are often wrong-headedly soft-hearted, to let children work, when their whole strength and energy should be concentrated on their education. This Act gave power to local authorities to decide as to the age up to which children must attend school. It is doubtful if local authorities are always the best qualified to decide such a question as this. They know the needs of the district, indeed, but, as a rule, they take more thought of its industrial than of its educational needs. Moreover, they can be "got at," not, indeed, by the child, whose welfare is in question, but by the rate-paying parent, who is as often as not more anxious to have his child earn wages than to have him obtain a good education. But more serious than this is a section of the Act, most clearly well intentioned, concerning the employment of children of school age, which does not fully calculate on the short-sighted selfishness of parents. This (section 4) runs as follows: "Every person who takes into his employment a child of the age of 10, and under the age of 13 years, resident in a district, before that child has obtained a certificate of having reached the standard of education fixed by a bye-law in force in the district for the total exemption of children of the like age from the obligation to attend school, shall be deemed to take such child into his employment in contravention of the Elementary Education Act, 1876, and shall be liable to a penalty accordingly."

This section fully protected children over ten; but it left those under that age to be exploited as their parents and employers chose! Moreover, certain excuses might be brought forward by the employer of child labour, and these were to be accepted as valid. The first, that there was not a school within two miles of the child's home, is not now often available. But the second is more serious. It permits employment, if this by reason of being during the school holidays, or during the hours during which school is not open, or otherwise, does not interfere with the efficient elementary instruction of such child, and if the child obtains such instruction by regular attendance for full time at a certified efficient school, or in some other equally efficient manner. The concession that a child may lawfully be employed at any time except during school hours obviously leaves great latitude. It is difficult to prove that the work done then may hinder the child from profiting by the instruction given at the school, and yet every teacher will avow that when a boy or girl has been at work for hours before coming to school, he is fit only to doze during school hours, and even if he can manage to keep awake his mind is too dull to profit by the instruction given. In what condition could that boy be for study who had been up all night in the dustyard? In short, any concession as to work in the case of children of school age is likely to be abused. The only hope lies in the complete disallowance of all exploiting of children's labour.

Not that even this would entirely do away with the evil. It might, indeed, prevent all outside work being done by children, and would save from themselves those who were trading on their own account (selling newspapers, flowers, &c.), as well as those who were working for parents or employers; but it is very difficult for the law to touch those home industries where the children of the family assist. When they are kept away from school for this purpose it is possible to impose a fine so large as to have a salutary effect on greedy parents; but it is difficult for the law to interfere with work done within the precincts of the home outside school hours. If it were tried we should have every extreme instance launched at our heads. Is a girl, for example, not to keep the baby after she comes home from school, or a boy to refuse to bring coals to the fire, for fear of the nervous exhaustion these tasks would entail? We know quite well that it is not this to which exception is taken; but the worshipper of "the god of things as they are" would be certain to talk as if these were the only possible tasks that could be assigned to a child.

